# Peri-Implantitis: Biofilm-Induced Disease or Prosthetically Driven Complication?

**DOI:** 10.7759/cureus.113497

**Published:** 2026-07-28

**Authors:** B. Roshan Arbaaz, S. Peter, S. Kangaiyan, Janarthanan Shiamala, Thangadurai Maheswaran, V. R Arunkumar

**Affiliations:** 1 Periodontics, Sri Venkateshwaraa Dental College, Puducherry, IND; 2 Prosthodontics, Sri Venkateshwaraa Dental College, Puducherry, IND; 3 Prosthodontics, Adhiparasakthi Dental College and Hospital, Melmaruvathur, IND; 4 Prosthodontics, Oral Safe Dental Clinic, Puducherry, IND; 5 Oral and Maxillofacial Pathology, Adhiparasakthi Dental College and Hospital, Melmaruvathur, IND

**Keywords:** biofilms, dental implants, emergence profile, interdisciplinary dentistry, occlusal overload, peri-implantitis, prosthesis design, risk factors

## Abstract

Peri-implantitis is a prevalent biological complication of implant therapy; however, whether it should be understood primarily as a biofilm-induced infection or as a consequence of prosthetically driven mechanical factors remains unclear. This narrative review synthesizes microbiological, biomechanical, and prosthetic evidence to address this question from an interdisciplinary perspective. Peri-implant biofilms occupy a distinct microbial niche, and their dysbiotic transition and the accompanying host response appear to be central to disease initiation, while an implant-directed foreign body reaction may contribute independently to bone loss. Occlusal overload, residual cement, and unfavorable emergence angles or contours do not independently initiate disease but consistently accelerate progression once inflammation is established, with wide emergence angles and cemented restorations with residual material showing particularly strong associations with the marginal bone loss. Epidemiological data confirm a multifactorial risk profile in which a history of periodontitis, smoking, and systemic conditions interact with local biofilms and prosthetic factors. Clinically, these findings support the integration of restorative, periodontal, and relevant endodontic assessments into implant treatment planning and long-term supportive care, as prosthetic design functions as a modifiable determinant of peri-implant health rather than a purely esthetic or mechanical consideration.

## Introduction and background

Dental implant therapy has become a routine and highly predictable option for replacing missing teeth. However, biological complications remain a significant and growing concern for clinicians across all specialties [1]. A dental implant restoration comprises the implant fixture, abutment, and prosthetic restoration. Successful osseointegration establishes direct structural and functional contact between bone and the implant surface. Unlike natural teeth, implants lack a periodontal ligament, resulting in distinct biomechanical behavior and peri-implant tissue architecture that influence disease development. Peri-implantitis, defined by the 2017 World Workshop as a plaque-associated pathological condition characterized by inflammation of the peri-implant mucosa and progressive loss of supporting bone relative to the post-restorative baseline [2], now affects an estimated one in four to five implant patients, with meta-analytic prevalence estimates ranging from approximately 21% to 25% at the patient level and a cumulative incidence approaching 22% over 20 years of function [3,4]. Peri-implant mucositis, a reversible precursor, is even more prevalent, affecting up to two of three patients in recent standardized estimates [3,4]. The economic and clinical burden is substantial; implant therapy is estimated to cost billions of dollars annually, even before accounting for the additional surgical, pharmacological, and maintenance costs generated by peri-implant complications [1].

Historically, peri-implantitis has been modeled as a bacterial disease analogous to periodontitis, and strong microbiological evidence supports a central etiological role for subgingival and submucosal biofilm dysbiosis [5,6]. However, this framing leaves important gaps. Comparative pathophysiological analysis indicates that peri-implantitis and periodontitis, despite sharing bacterial drivers, are histologically and clinically distinct [7]. Some authors have proposed that a foreign body immune response to titanium implants may contribute independently to the marginal bone loss [1]. Simultaneously, a substantial and partly separate body of evidence implicates occlusal overload, cement retention technique, and unfavorable prosthetic emergence geometry as risk factors whose contribution is difficult to reconcile with a purely microbial model [8]. Established risk indicators for peri-implantitis span the microbiological, behavioral, and mechanical domains without a unifying etiological consensus [3,8].

This apparent divide between biofilm-induced disease and prosthetically driven complications has rarely been examined as a single integrated question spanning periodontology, prosthodontics, and restorative dentistry. The present narrative review addresses this gap by critically appraising the microbiological, biomechanical, and prosthetic determinants of peri-implantitis side by side, in order to clarify how these domains interact and to inform a genuinely interdisciplinary approach to prevention and management.

## Review

Methodology

Literature for this narrative review was identified through targeted PubMed searches using combinations of terms related to peri-implantitis, peri-implant mucositis, dental implants, biofilm, microbiome, prosthetic design, emergence profile, emergence angle, occlusal overload, residual cement, marginal bone loss, and peri-implant risk factors. PubMed was selected as the primary database because of its comprehensive coverage of peer-reviewed biomedical and dental literature. English-language publications were preferentially selected, with emphasis on recent evidence while including landmark studies where appropriate. References were selected based on relevance to the review objectives and included systematic reviews, meta-analyses, clinical investigations, consensus statements, and international clinical practice guidelines. Owing to the narrative nature of this review, no predefined systematic search protocol, protocol registration, or formal risk-of-bias assessment was performed.

Biofilm-driven etiopathogenesis: microbiology and host response

The peri-implant microbiome constitutes a distinct ecological niche rather than a simple extension of the periodontal environment. Comparative 16S ribosomal RNA (rRNA) gene sequencing has shown that peri-implant bacterial communities harbor significantly lower taxonomic diversity than periodontal communities in both health and disease, with several genera occurring almost exclusively around implants [5]. Even within the same patient, adjacent peri-implant and periodontal sites can share less than a tenth of their abundant bacterial species, underscoring the relative independence of the peri-implant ecosystem [5]. As the disease progresses, this niche undergoes a characteristic transition from health to peri-implantitis, marked by a gradual increase in community diversity, depletion of commensal taxa, and enrichment of both classical periodontal pathogens and emerging, less-studied organisms such as staphylococci [5]. Metatranscriptomic analyses have further revealed that, although peri-implantitis and periodontitis biofilms share overlapping virulence pathways, they diverge in specific metabolic routes and interbacterial networks, arguing against a purely interchangeable disease model [5].

Severity-linked profiling reinforces the etiological weight of submucosal dysbiosis. Full-length 16S rRNA sequencing combined with metatranscriptomics of implants diagnosed with peri-implantitis has demonstrated that specific taxa and functional pathways correlate quantitatively with probing depth, a proxy for disease severity, allowing the proposal of a composite microbial dysbiosis index that integrates taxonomic and enzymatic features [6]. In this framework, established periodontopathogens dominate irrespective of pocket depth, whereas other taxa track more closely with deepening pockets, suggesting that some organisms mark chronic dysbiosis rather than actively driving tissue destruction [6]. Functional predictions additionally link shifts in nitrate reduction, sulfate assimilation, and related metabolic pathways to increasing severity, extending the disease model beyond a simple taxonomic description toward a mechanistic, activity-based understanding of peri-implant biofilms [6]. Current evidence suggests that disease is initiated by dysbiotic biofilm rather than a single trigger microorganism. Although peri-implantitis shares several periodontal pathogens with periodontitis, differences in the peri-implant ecological niche and host response explain their distinct biological behavior while accounting for the increased susceptibility of patients with a history of periodontitis.

However, this microbiological picture does not fully answer the question of primary causation. A narrative synthesis of contemporary pathogenesis concepts notes that although gram-negative anaerobic biofilms remain central to disease initiation, the foreign-body response elicited by the titanium surface may represent a parallel, non-infectious contributor to marginal bone loss [1]. This has been proposed as a complementary and still debated mechanism that may contribute to disease progression alongside biofilm-induced inflammation, although current evidence continues to support biofilm dysbiosis as the primary etiological driver. Under this reframing, osseointegration is conceptualized as a foreign-body reaction held in equilibrium with the host, mediated by complement activation and macrophage polarization, such that bone loss may reflect a breakdown of this immune equilibrium rather than bacterial destruction alone [1]. Comparative pathophysiological analysis supports the view that, despite sharing bacterial etiology and clinical features with periodontitis, peri-implantitis constitutes a histologically distinct entity, producing larger inflammatory infiltrates and more pronounced tissue destruction for a comparable microbial challenge [7]. Together, these findings frame peri-implantitis as a condition in which biofilm accumulation is a necessary trigger, while the resulting host response, shaped by the unique peri-implant anatomy and implant material, determines the ultimate pattern and severity of tissue breakdown.

Occlusal overload and biomechanical complications

Because dental implants lack a periodontal ligament and its associated mechanoreceptive feedback, they cannot modulate occlusal forces as natural teeth do, leaving the peri-implant bone vulnerable to biomechanical trauma [9]. A systematic review synthesizing 80 studies found that marginal bone loss attributable to occlusal factors ranged from approximately 0.65 to 1.20 mm, while more overt traumatic occlusal forces were associated with bone level changes of 1.0 to 3.0 mm and peri-implantitis incidence of 20% to 50% [9]. Parafunctional habits, such as bruxism, intensify these effects, and the review explicitly highlighted a synergistic relationship between mechanical overload and bacterial biofilms in accelerating peri-implant tissue breakdown, rather than treating the two as independent pathways [9].

Earlier systematic evidence has anticipated this interaction. Animal studies assessing supra-occlusal contacts on osseointegrated implants found that overload alone, in the absence of peri-implant inflammation, did not compromise osseointegration and was anabolic to bone, whereas the same loading in an inflamed environment significantly aggravated plaque-induced bone resorption [10]. Consistent with this, a further review concluded that strong evidence links occlusal factors to mechanical complications such as screw loosening or fracture, but that evidence for a direct, independent biological effect on peri-implant tissue is far weaker; occlusal overload appears instead to function as an accelerating cofactor once inflammation is already present [11]. An earlier clinical review focusing specifically on biomechanical complications similarly identified occlusal overloading as the primary cause of implant fixture and prosthetic component fracture or loosening and reported a positive association with peri-implant marginal bone loss across the studies examined, while cautioning that the underlying evidence base remained limited to a small number of eligible clinical reports [12]. Collectively, these findings indicate that occlusal overload is not an independent cause of peri-implantitis but a biomechanical amplifier that interacts with and may accelerate an underlying inflammatory biofilm process.

Cement retention and prosthetic attachment-related risk

The choice between cement- and screw-retained restorations remains one of the most debated prosthetic decisions in implant dentistry, and endoscopic evidence has clarified how residual cement contributes to diseases. A cross-sectional endoscopic study of patients with peri-implant disease around cement-retained crowns found submucosal cement residues in 80.4% of patients, concentrated in the vestibular and lingual areas, and showed that the apical position of the residue was significantly deeper in peri-implantitis cases than in mucositis cases, directly linking the spatial extent of cement contamination to disease severity [13]. The same body of literature identifies two non-exclusive etiopathogenic routes for this effect: a direct route in which residual cement acts as a foreign body or chemical irritant provoking local cytokine-mediated bone resorption, and an indirect route in which the rough, retentive cement surface promotes bacterial adhesion and pathogenic biofilm formation [13].

Comparative clinical data help contextualize cement-specific risks against alternative screw-retained approaches. A retrospective cohort study comparing 38 cemented and 38 screw-retained single-implant crowns over 24 months found significantly greater marginal bone loss with cemented restorations (1.12 ± 0.34 mm versus 0.68 ± 0.29 mm), with excess cement occurring in 34% of cemented cases and correlating strongly with the degree of bone loss [14]. An earlier retrospective case analysis similarly found peri-implant disease in 85% of implants with cement remnants compared with only 1.08% of screw-retained controls and noted that implants in patients with a history of periodontitis uniformly developed peri-implantitis when cement remnants were present [15]. However, the apparent superiority of screw retention is not absolute at the aggregate level. A large systematic review and meta-analysis of prosthetic design parameters found no statistically significant difference in marginal bone loss between screw-retained and cement-retained prostheses, while identifying nonsplinted restorations, platform-switched abutments, internal (particularly conical) implant-abutment connections, abutment heights of ≥2 mm, and a one-abutment-one-time protocol as the design features most consistently associated with reduced bone loss [16]. This divergence suggests that excess undetected cement, rather than the cementation technique, drives the elevated risk observed in individual cohort studies. Despite these risks, cement-retained restorations remain widely used because of their favorable esthetics, passive fit, and simplified occlusal design. Careful cementation protocols and meticulous removal of excess cement are therefore essential to minimize biological complications.

Emergence profile and prosthetic contour design

The restorative emergence profile shapes the biological seal around an implant and, through it, the accessibility of the site to hygiene. The implant supracrestal complex comprises approximately 2 mm of epithelial attachment and 1.5 to 2 mm of connective tissue, forming a protective zone of approximately 3.5 to 4 mm above the implant platform that lacks the perpendicular collagen anchorage present around natural teeth, leaving it comparatively vulnerable to biofilm-driven inflammation when the contour is unfavorable [17]. A narrative review integrating histological and clinical evidence found that concave, polished emergence profiles supported by taller titanium bases (≥2 mm) preserve this biological seal and promote soft-tissue stability, whereas mucosal tunnels exceeding 3 mm are associated with elevated plaque accumulation and delayed resolution of inflammation even under regular maintenance, and illustrated the point with a case in which redesigning a convex restoration into a concave, polished contour resolved established peri-implant inflammation without additional surgery [17].

A critical review of the implant supracrestal complex similarly concluded that a wide emergence angle combined with a convex emergence profile is linked to peri-implantitis and that modifying the restoration contour is an effective adjunct to the anti-infective treatment of peri-implant mucositis, although evidence regarding abutment or prosthesis material and retention type remains inconclusive [18]. Quantitative support comes from a retrospective radiographic study in which implant malposition itself, defined by established spatial criteria, was not significantly associated with peri-implantitis, but a prosthetic emergence angle of 45° or more carried an odds ratio of 9.094 for peri-implantitis, a magnitude of association exceeding even that of a prior history of periodontitis (odds ratio 5.945) in the same model [19]. A broader comprehensive review likewise reported that emergence angles exceeding 30° and convex prosthetic designs increase the risk of peri-implantitis and bone resorption in several, though not all, studies, while taper or conical abutment-implant joint connections consistently favor marginal bone stability, reinforcing cleanability as the unifying clinical priority across differing implant systems [20]. Table 1 summarizes the principal prosthetic and mechanical factors implicated in peri-implant marginal bone loss and peri-implantitis risk across the studies reviewed above. Most available evidence is observational; therefore, these prosthetic characteristics should be interpreted as important risk modifiers and associations rather than definitive causal determinants.

**Table 1:** Prosthetic and mechanical factors associated with peri-implant marginal bone loss and peri-implantitis risk Source: Original table created by the authors based on data synthesized from references [9, 13-20]. Reported estimates are derived either from pooled meta-analyses/systematic reviews or individual observational studies, as indicated by the corresponding references. Abbreviations: MBL, marginal bone loss; OR, odds ratio.

Prosthetic/mechanical factor	Comparison	Reported effect on MBL/peri-implantitis risk	Source(s)
Occlusal loading	Traumatic/excessive vs physiological	MBL 0.65–3.0 mm; peri-implantitis incidence 20%-50%	[9]
Retention type	Cemented vs screw-retained	No significant pooled MBL difference overall; cemented cohorts with excess cement show higher MBL (1.12 ± 0.34 vs 0.68 ± 0.29 mm)	[14,16]
Residual/excess cement	Present vs absent	Peri-implant disease in 85% of cemented implants with residues vs 1.08% of screw-retained controls; residues detected in 80.4% of diseased implants, more apical in peri-implantitis	[13,15]
Emergence angle	≥45° (or >30°) vs narrower	OR 9.094 for peri-implantitis; increased risk in most but not all studies	[19,20]
Emergence profile contour	Convex vs concave/polished	Convex contour linked to peri-implantitis; concave, polished contour promotes soft-tissue stability	[17,18]
Abutment–implant connection	External vs internal/conical	Internal, particularly conical, connections associated with reduced MBL	[16,20]
Abutment height	<2 mm vs ≥2 mm	Taller abutments (≥2 mm) associated with reduced MBL	[16,17]
Implant splinting	Splinted vs nonsplinted	Nonsplinted restorations show lower MBL	[16]
Abutment disconnection protocol	Repeated vs one-abutment-one-time	Repeated disconnections associated with greater MBL	[16]

**Figure 1:**
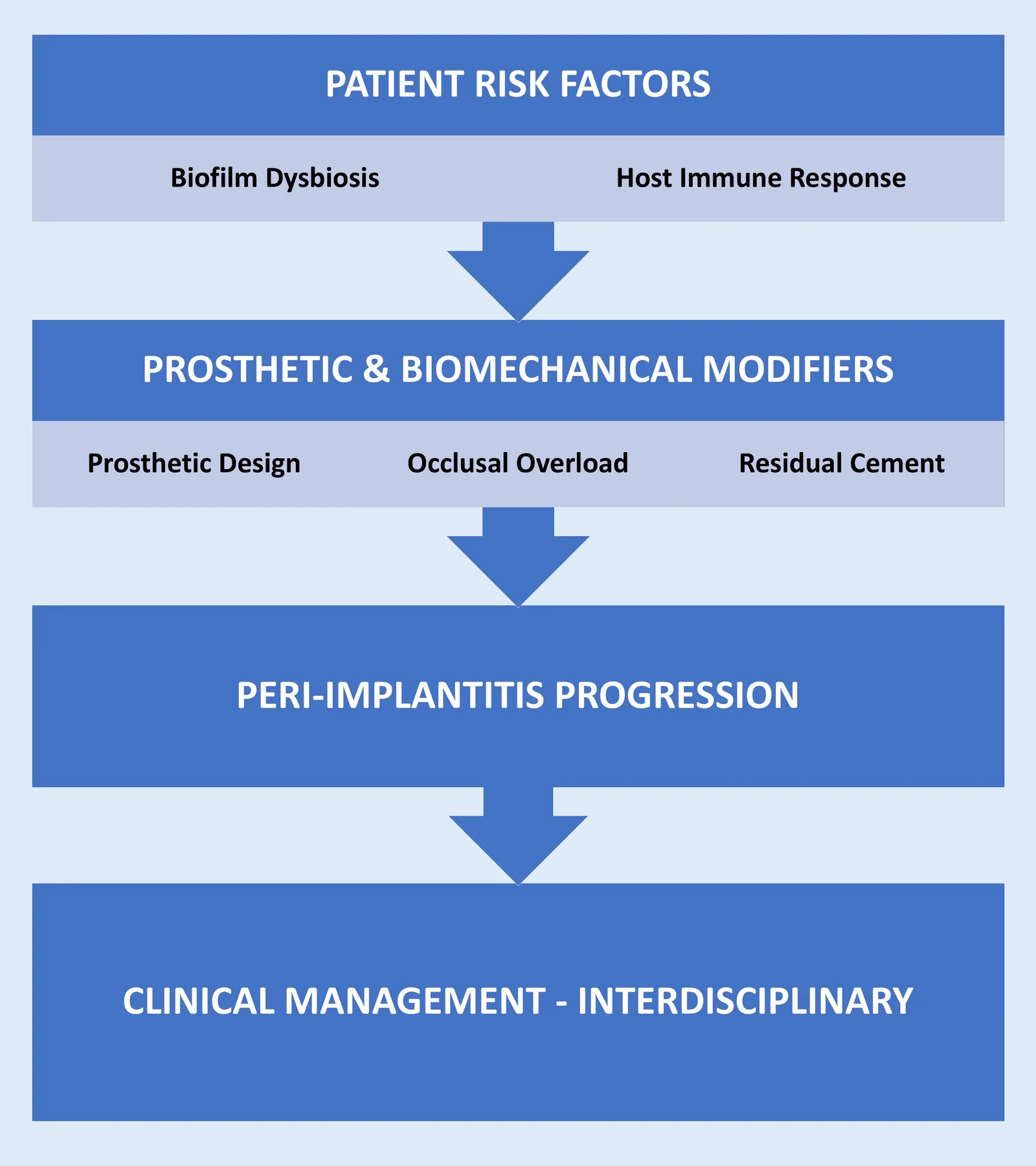
Conceptual model of peri-implantitis Conceptual model illustrating the interaction between biofilm dysbiosis, host immune response, prosthetic design, biomechanical loading, and peri-implant disease progression.

A conceptual model illustrating the interaction between biofilm dysbiosis, host immune response, prosthetic design, biomechanical loading, and peri-implant disease progression is presented in Figure 1.

Epidemiology and multifactorial risk indicators

Peri-implant diseases are common in implant dentistry, although estimates vary depending on the case definition. The 2017 World Workshop on the Classification of Periodontal and Peri-Implant Diseases and Conditions defined peri-implantitis as a pathological plaque-associated condition characterized by inflammation of the peri-implant mucosa and progressive loss of the supporting bone [21]. They concluded that progressive crestal bone loss in the complete absence of soft-tissue inflammation is a rare event, reinforcing the centrality of an inflammatory biofilm-associated process to the disease definition itself [2]. Using this standardized framework, a systematic review restricted to studies applying the 2017 criteria estimated a weighted mean prevalence of peri-implant mucositis of 63% at the patient level and of peri-implantitis of 25%, with prevalence varying significantly by continent and dropping sharply to 5.2% at the implant level among non-smokers [4]. Reported prevalence estimates should be interpreted in the context of differing case definitions and methodological heterogeneity among studies. 

A larger meta-analysis spanning 102 studies and more than 13,000 patients estimated the patient-level prevalence at 46% for mucositis and 21% for peri-implantitis, with corresponding 20-year cumulative incidences of 53% and 22%, respectively. Periodontitis, smoking, diabetes mellitus, and alcohol consumption have been identified as significant systemic and behavioral risk indicators for peri-implantitis, whereas obesity has been identified as a risk factor for mucositis [3]. These figures sit within, although at the higher end, of earlier pooled estimates. A narrative review of risk indicators similarly concluded that a history of periodontal disease, smoking, excess cement, and lack of regular supportive maintenance should all be regarded as risk indicators for peri-implantitis, while noting preliminary evidence implicating genetic polymorphisms and diabetes mellitus [8]. Taken together, this epidemiological evidence establishes that peri-implant disease is a multifactorial condition in which patient-level susceptibility, chiefly periodontal history and smoking, interacts with local biofilm accumulation to determine both the probability and severity of disease onset, rather than either factor alone providing a sufficient explanation.

Diagnosis, prevention, and interdisciplinary management

Because peri-implant disease arises at the intersection of microbiology, biomechanics, and prosthetic design, its prevention and management are explicitly framed as interdisciplinary tasks in the current clinical guidelines. The European Federation of Periodontology S3-level clinical practice guidelines recommend implementing preventive interventions from the point of treatment planning through surgical placement and prosthetic loading, followed by a structured, risk-stratified supportive peri-implant care program once the restoration is in function, and these recommendations were derived from 13 commissioned systematic reviews synthesized through a formal consensus process [22]. These guidelines additionally emphasize individualized risk assessment, patient education, prosthetic maintenance, and structured supportive peri-implant care as integral components of disease prevention [22]. An earlier consensus report focused specifically on primary prevention and similarly emphasized that regular supportive therapy and mechanical plaque control are the most effective preventive measures against the conversion of peri-implant mucositis to peri-implantitis, whereas adjunctive antiseptics or antibiotics provided no proven benefit beyond mechanical debridement [23].

The interdisciplinary dimension extends beyond periodontal maintenance to include restorative and endodontic domains. A systematic review of retrograde peri-implantitis, a form of disease originating apically rather than at the marginal biofilm interface, found a higher occurrence when implants were placed near periapical lesions of adjacent teeth, close to a periodontitis-affected extraction site, or in close proximity to a neighboring tooth. It has been reported that surgical treatment of the affected implant combined with endodontic management of the adjacent tooth yielded survival rates as high as 100%, directly implicating restorative planning and endodontic status as co-determinants of implant outcomes [24]. Diagnostic innovation is likewise reframing management: conventional clinical and radiographic parameters cannot reliably predict future disease activity or implant failure, prompting the development of molecular point-of-care tests capable of detecting active tissue destruction from crevicular fluid before clinical signs appear, which may allow earlier and more targeted intervention when integrated with traditional staging and grading protocols [25]. Roccuzzo et al. have highlighted the impact on the soft tissue handling associated with bone reconstructive procedures and on the importance of soft tissue conditions to enhance and maintain peri-implant health in the long-term [26]. Collectively, these findings support a management model in which no single specialty (periodontal, prosthetic, or endodontic) can independently prevent or resolve peri-implant disease. Therefore, current evidence supports individualized treatment combining non-surgical biofilm control, surgical intervention when indicated, prosthetic modification, and long-term supportive maintenance, reflecting the multifactorial nature of peri-implantitis. Emerging artificial intelligence-assisted radiographic analysis and predictive diagnostic models may further improve early detection and longitudinal monitoring of peri-implant disease, although clinical validation remains ongoing.

Limitations

This review is subject to the inherent limitations of narrative reviews. The literature search was limited to the PubMed database, and study selection was based on author judgement rather than predefined systematic eligibility criteria. Restriction to the PubMed database may have introduced selection bias through omission of studies indexed exclusively in other databases such as Embase or Scopus. No formal assessment of study quality or risk of bias was performed, and no quantitative evidence synthesis was performed. Consequently, the findings should be interpreted as a qualitative synthesis of the available evidence, rather than a comprehensive systematic evaluation.

## Conclusions

The evidence reviewed here indicates that peri-implantitis cannot be reduced to either a purely biofilm-induced infection or a purely prosthetically driven mechanical complication; it is more accurately understood as a biofilm-initiated disease whose onset, severity, and rate of progression are substantially modulated by prosthetic and biomechanical factors. Microbial dysbiosis and an aberrant host response, potentially compounded by the foreign-body component of osseointegration, appear to be necessary for disease initiation, while occlusal overload, cement retention technique, and unfavorable emergence geometry function as accelerants that determine how readily an inflamed site progresses to bone loss. For clinical practice, this synthesis translates into a single actionable principle: prosthetic design decisions, including retention type, emergence angle and contour, abutment height, and connection geometry, should be treated as active risk-modifying interventions rather than purely restorative or esthetic choices, and should be planned jointly by the restorative and surgical teams alongside conventional periodontal risk assessment, supportive maintenance, and, where relevant, endodontic evaluation of adjacent teeth. Future research should prioritize well-designed prospective studies, standardized case definitions, and interdisciplinary investigations clarifying the biological interactions between biofilm dysbiosis, host response, and prosthetic factors.

## References

[1] Fragkioudakis I, Tseleki G, Doufexi AE, Sakellari D (2021). Current concepts on the pathogenesis of peri-implantitis: a narrative review. Eur J Dent.

[2] Schwarz F, Derks J, Monje A, Wang HL (2018). Peri-implantitis. J Periodontol.

[3] Galarraga-Vinueza ME, Pagni S, Finkelman M, Schoenbaum T, Chambrone L (2025). Prevalence, incidence, systemic, behavioral, and patient-related risk factors and indicators for peri-implant diseases: an AO/AAP  systematic review and meta-analysis. J Periodontol.

[4] Reis IN, Huamán-Mendoza AA, Ramadan D (2025). The prevalence of peri-implant mucositis and peri-implantitis based on the world workshop criteria: a systematic review and meta-analysis. J Dent.

[5] Belibasakis GN, Manoil D (2021). Microbial community-driven etiopathogenesis of peri-implantitis. J Dent Res.

[6] Joshi AA, Szafrański SP, Steglich M (2026). The submucosal microbiome correlates with peri-implantitis severity. J Dent Res.

[7] Salvi GE, Stähli A, Imber JC, Sculean A, Roccuzzo A (2023). Physiopathology of peri-implant diseases. Clin Implant Dent Relat Res.

[8] Renvert S, Quirynen M (2015). Risk indicators for peri-implantitis. A narrative review. Clin Oral Implants Res.

[9] Mojaver S, Patel N, Sarmiento H, Fiorellini JP (2025). Under pressure: Unraveling the impact of occlusal overload on peri-implant health-a systematic review. J Prosthodont.

[10] Naert I, Duyck J, Vandamme K (2012). Occlusal overload and bone/implant loss. Clin Oral Implants Res.

[11] Lee SJ, Alamri O, Cao H, Wang Y, Gallucci GO, Lee JD (2023). Occlusion as a predisposing factor for peri-implant disease: a review article. Clin Implant Dent Relat Res.

[12] Fu JH, Hsu YT, Wang HL (2012). Identifying occlusal overload and how to deal with it to avoid marginal bone loss around implants. Eur J Oral Implantol.

[13] Montevecchi M, Valeriani L, Salvadori MF, Stefanini M, Zucchelli G (2025). Excess cement and peri-implant disease: a cross-sectional clinical endoscopic study. J Periodontol.

[14] C R V, Barodiya A, Gaikwad A, C B SL, Kumar S, Shah M (2026). Comparative study of cemented versus screwed prosthesis on the marginal bone stability around dental implants. Bioinformation.

[15] Linkevicius T, Puisys A, Vindasiute E, Linkeviciene L, Apse P (2013). Does residual cement around implant-supported restorations cause peri-implant disease? A retrospective case analysis. Clin Oral Implants Res.

[16] Lin GH, Lee E, Barootchi S, Rosen PS, Curtis D, Kan J, Wang HL (2025). The influence of prosthetic designs on peri-implant bone loss: An AO/AAP systematic review and meta-analysis. J Periodontol.

[17] Pirc M, Esquivel J, Patrizzi A, Jung RE, Strauss FJ (2026). Emergence profile angle matters-restoring peri-implant health by adjusting prosthetics-a narrative review. J Esthet Restor Dent.

[18] Mattheos N, Janda M, Acharya A, Pekarski S, Larsson C (2021). Impact of design elements of the implant supracrestal complex (ISC) on the risk of peri-implant mucositis and peri-implantitis: a critical review. Clin Oral Implants Res.

[19] Corbella S, Morandi B, Calciolari E, Alberti A, Francetti L, Donos N (2023). The influence of implant position and of prosthetic characteristics on the occurrence of peri-implantitis: a retrospective study on periapical radiographs. Clin Oral Investig.

[20] Izzetti R, Cinquini C, Nisi M, Covelli M, Alfonsi F, Barone A (2025). Influence of prosthetic emergence profile on peri-implant marginal bone stability: a comprehensive review. Medicina (Kaunas).

[21] Berglundh T, Armitage G, Araujo MG (2018). Peri-implant diseases and conditions: Consensus report of workgroup 4 of the 2017 World Workshop on the Classification of Periodontal and Peri-Implant Diseases and Conditions. J Periodontol.

[22] Herrera D, Berglundh T, Schwarz F (2023). Prevention and treatment of peri-implant diseases-the EFP S3 level clinical practice guideline. J Clin Periodontol.

[23] Jepsen S, Berglundh T, Genco R (2015). Primary prevention of peri-implantitis: managing peri-implant mucositis. J Clin Periodontol.

[24] Sinsareekul C, Limpuangthip N (2025). Potential risks, treatment strategies, and treatment outcomes of retrograde peri-implantitis: a systematic review. J Prosthet Dent.

[25] Bornes R, Montero J, Correia A, Marques T, Rosa N (2023). Peri-implant diseases diagnosis, prognosis and dental implant monitoring: a narrative review of novel strategies and clinical impact. BMC Oral Health.

[26] Roccuzzo M, Roccuzzo A, Marruganti C, Fickl S (2023). The importance of soft tissue condition in bone regenerative procedures to ensure long-term peri-implant health. Periodontol 2000.

